# Comprehensive prognostic and immunological analysis of CCT2 in pan-cancer

**DOI:** 10.3389/fonc.2022.986990

**Published:** 2022-09-01

**Authors:** Wenming Lv, Lin Shi, Jiebing Pan, Shengbao Wang

**Affiliations:** ^1^ Department of Neurology, Second Hospital of Lanzhou University, Lanzhou, China; ^2^ Department of Hematology, Peking University International Hospital, Beijing, China; ^3^ Department of General Surgery, Lanzhou University Second Hospital, Lanzhou, China; ^4^ Emergency Center of the Second Hospital of Lanzhou University, Lanzhou, China

**Keywords:** CCT2, pan-cancer, prognosis, immune infiltration, tumor microenvironment

## Abstract

CCT2 acts as a molecular chaperone protein that assists in the proper folding of proteins, thus ensuring a dynamic balance of cellular homeostasis. Despite increasing evidence supporting the important role of CCT2 in the tumorigenesis of certain cancers, few articles that provide a systematic pan-cancer analysis of CCT2 have been published. Hence, to evaluate the expression status and prognostic significance of CCT2 in pan-cancers, an analysis of the relationship between CCT2 and different tumor immune cell infiltrations was conducted using datasets from the Cancer Genome Atlas, Cancer Cell Lineage Encyclopedia, and so on. In most cancers, CCT2 expression was high and was associated with poor prognosis. Moreover, CCT2 gene expression was negatively correlated with infiltration of most immune cells in 10 cancer types, and CCT2 expression was related to tumor mutation burden and microsatellite instability. The role that CCT2 plays in tumorigenesis and tumor immunity suggests that it can serve as a prognostic marker in many cancers.

## Introduction

Chaperone proteins are a family of proteins that wrap around a substrate and help it to fold in an ATP-dependent manner and are classified into group I (heat shock protein 60 or GroEL) and group II (TCP-1 chaperone protein complex) (1). CCT is an important molecule for the synthesis of TCP-1 ring-containing chaperone protein complex (TRiC) with two ring structures (2). One-tenth of the proteins in cells are folded by TRiC, including actin and microtubulin, and it regulates the expression of tumor-associated proteins and cell cycle, which are aberrantly expressed in many tumors and are potential targets for treatment (3). CCT plays a role in the binding and hydrolysis of ATP as well as the recognition and folding of substrates as part of TRiC (4, 5). There are eight different subunits (CCT1–CCT8), each of which has different substrate recognition and ATP hydrolysis properties (6).

The CCT family of genes is closely associated with the development of tumors according to several studies (7, 8). Hepatocellular carcinoma, for example, exhibits high levels of TCP1/CCT2-CCT8 expression and low levels of CCT6B, which leads to the abnormal regulation of Myc target genes and hypoxia-inducible factor target genes as well as cell cycle abnormalities (3). A study by Carr et al. (9) examined the effects of the CCT family genes on the development of hepatocellular carcinoma by examining the protein levels of CCT subunits in hepatocellular carcinoma, prostate cancer, and lung cancer, where higher levels of CCT2 were detected than in normal tissue. In gallbladder cancer, positive CCT2 expression was negatively correlated with low postoperative patient survival and positively correlated with high mortality (10). In our previous study, we found that CCT2 expression showed an increasing trend in normal, ulcerative colitis, and colon cancer tissues by analyzing data from The Cancer Genome Atlas (TCGA) database and Gene Expression Omnibus (GEO) database and predicted the possibility that CCT2 may be closely associated with the development of colon inflammation or cancer and that CCT2 is a prognostic factor for colon cancer (11). Therefore, a combination of previous studies and our previous study started from CCT2 and cited the clinical data of various human cancers, genomic variants, mRNA expression, miRNA expression, methylation, and other data included in TCGA database as well as protein expression in different human tissues and organs included in the Human Protein Atlas (HPA) database in order to explore the function of the role CCT2 plays in various cancers. The aim is to investigate the function of CCT2 in various cancers.

In summary, CCT2 expression has been shown to be related to poor prognosis in numerous types of tumors. In distinction, most reports to this point are restricted to investigations on the role of CCT2 in specific styles of cancer. Pan-cancer research on the association between CCT2 and varied cancers has not been reported. Therefore, we tend to analyze the CCT2 expression levels and their association with the prognosis of various styles of malignancies supported by multiple databases of TCGA, HPA, and so on.

## Methods

### Data sources

Gene data in tumors and normal tissues were obtained from The Cancer Genome Atlas database and the Genotype–Tissue Expression project database, and the data for tumor cell lines were from the Cancer Cell Line Encyclopedia database. TCGA (The Cancer Genome Atlas, www.cancer.gov) database was used for the expression differential analysis and validation of CCT2 expression correlation with clinical features, HPA (www.proteinatlas.org) database was used to detect CCT2 protein expression analysis, xCell database was used for the association of CCT2 expression with immune cell infiltration, and GEO database was used to download and analyze the transcriptome data of colon cancer GSE143985 and thyroid cancer GSE33630.

### CCT2 expression analysis

The tumor data were obtained from the TCCGA database, the paraneoplastic data were obtained from the TCGA dataset, and the expression differences of various tumors were analyzed by R software. The expression levels of CCT2 in different tissues and different tumor tissues were analyzed using the Kruskal–Wallis test.

### Prognostic analysis in pan-cancer

One-way Cox multivariate analysis was utilized to calculate the correlation between CCT2 expression and patient survival in 33 tumors, and Kaplan–Meier survival analysis was employed to compare the link between high and low CCT2 expression levels and tumor prognosis.

### Correlation analysis of immune microenvironment

The correlation between CCT2 expression and immune cell scores was analyzed by downloading the scores of six immune infiltrating cells from the xCell for 33 styles of cancer. The immune score and the stromal score of every tumor sample were analyzed using the R software package ESTIMATE to determine the correlation between CCT2 expression and immune score in 33 tumors. The correlation was considered significant and positive once *p <*0.05 and *R >*0.20.

### Immunohistochemical staining

Immunohistochemistry (IHC) pictures of CCT2 protein expression in normal and tumor tissues were downloaded from HPA to conduct a differential analysis of CCT2 expression at the protein level.

### Analysis of the relationship between CCT2 and clinical phenotype

Patient survival and clinical phenotype data were uploaded from the TCGA database. Two clinical phenotypes—tumor staging and grading—were selected, and their relationship with CCT2 expression was analyzed using the R package “limma” and “ggpubr”, the result of which was considered significant at *p <*0.05.

### Correlation of CCT2 expression with tumor mutation burden and tumor microsatellite instability

The tumor mutation burden (TMB) scores were calculated using Perl scripts and corrected by dividing by the full length of exons. The MSI scores were determined for all samples, supported with corporal mutation data obtained from TCGA and an analysis of the link between CCT2 expression and TMB and MSI using Spearman’s rank coefficient of correlation. Therefore, the resulting square measurements, shown as heat maps, using the R package “reshape2” and “RColorBrewer” were generated.

### Significance of CCT2 expression

Gene set enrichment analysis (GSEA) was performed to study the function of CCT2 in tumors. The Gene Ontology and Kyoto Encyclopedia of Genes and Genomes (KEGG) gene sets were downloaded from the official GSEA website, and functional analysis was performed using the R package “limma”.  

### RT-qPCR

Total RNA was isolated from cultured cells using Trizol reagent (Invitrogen) according to the instructions. One microgram of RNA was reverse-transcribed to cDNA using PrimerScript RT Master Mix (Takara, Dalian, China). RT-qPCR experiments were then performed according to the instructions using SYBR Premix Ex Taq (Takara, Dalian, China) on ABI 7500 (ABI, America) for qRT-PCR experiments, with GAPDH as an internal reference, and the relative expression of genes was calculated by the 2^-△△Ct^ method, respectively. The PCR primers are shown in **Table 1**.

**Table 1:** Primer sequence for RT-qPCR.

Gene	Primer sequence	
CCT2	Forward	5’-GCACTACCTCTGTTACCGTTTT-3’
	Reverse	5’-CTTCTCTCCAACCCGCTATGA-3’
GAPDH	Forward	5’-CGGAGTCAACGGATTTGGTCGTAT-3’
	Reverse	5’-AGCCTTCTCCATGGTGGTGAAGAC-3’

## Results

### CCT2 pan-cancer expression

**Figure 1:**
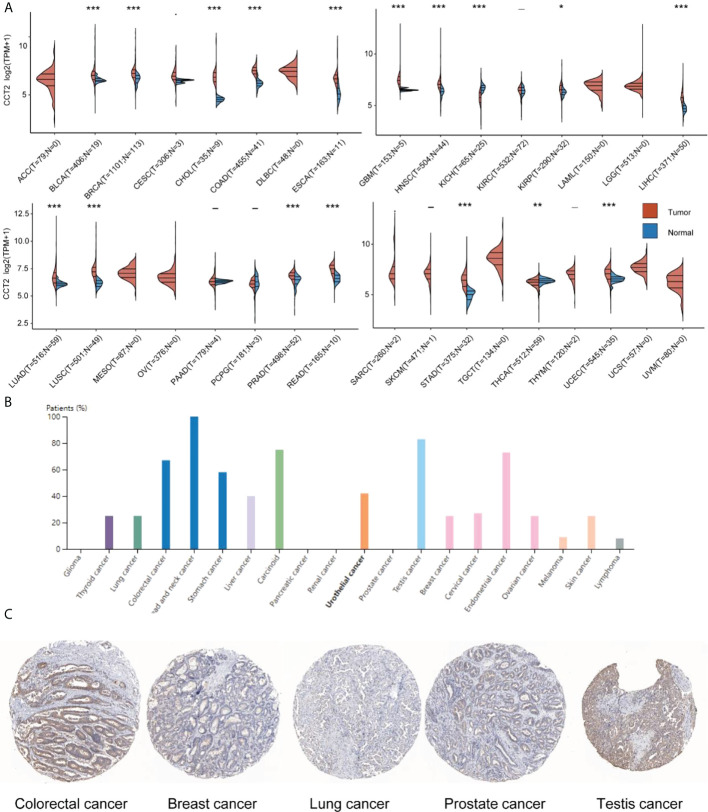
CCT2 expression in human pan-cancer. **(A)** Differential expression of CCT2 mRNA in tumor and normal tissues in the TCGA cohort. **(B)** Twenty cancer types in which CCT2 protein pachytene was present. **(C)** Representative immunohistochemical staining for CCT2 in Human Protein Atlas. *p < 0.05, **p < 0.01, ***p < 0.001.

Compared with normal sample tissues, CCT2 was expressed in BLCA, BRCA, CHOL, CESC, COAD, ESCA, GBM, HNSC, KIRP, LIHC, LUAD, LUSC, PRAD, READ, STAD, and UCEC, which was upregulated in LAML and downregulated in KICH and THCA (**Figure 1A**). In addition, we also investigated CCT2 protein expression in the HPA cohort, which was expressed at varying degrees in all but glioma, pancreatic, renal, and prostate cancers (**Figure 1B**), a representative immunohistochemical map of which is shown in **Figure 1C**.

### The prognostic value of CCT2

**Figure 2:**
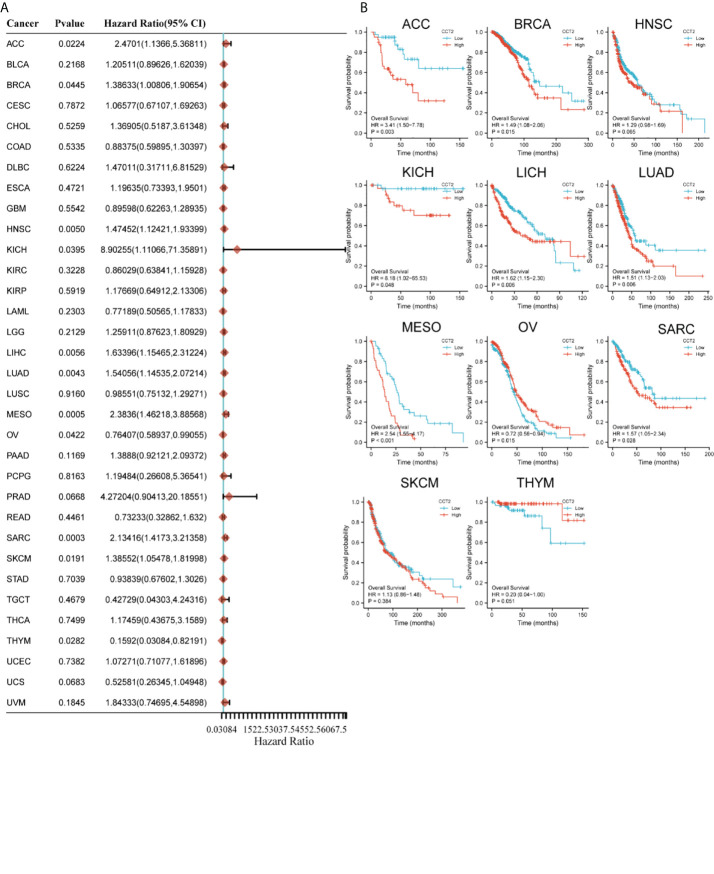
Relationship between CCT2 expression and overall survival time. **(A)** Forest plot of overall survival (OS) association of 33 tumors. **(B)** Kaplan–Meier analysis of CCT2 expression and OS.

The Cox regression analysis figured out that CCT2 expression was related to OS in 11 cancers: ACC, BRCA, HNSC, KICH, LICH, LUAD, MESO, OV, SARC, SKCM, and THYM (**Figure 2A**). The Kaplan–Meier survival curves showed that the CCT2 expression levels were significantly associated with prognosis in ACC, BRCA, KICH, LICH, LUAD, MESO, OV, SARC, and THYM, where a high CCT2 expression was associated with poor prognosis in cancers other than OV (**Figure 2B**).

### Pan-cancer analysis of CCT2 expression and clinicopathological correlation

**Figure 3:**
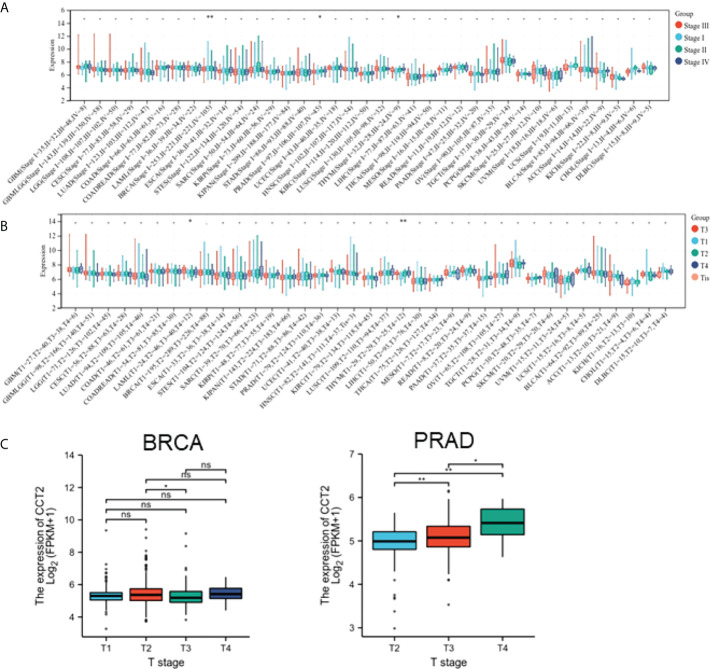
Association between CCT2 expression and clinical characteristics. **(A)** Association between CCT2 expression and tumor stage grading. **(B)** Association between CCT2 expression and BRCA and PRAD tumor stages. **(C)** Expression of BRCA and PRAD in different tumor stages. *p < 0.05, **p < 0.01. NS, no significant.

To explore the correlation between CCT2 expression and the clinicopathological features of cancer, we evaluated CCT2 expression in patients with different grades and stages of cancer. The results revealed that CCT2 expression in BRCA, PRAD, and THYM was significantly different from tumor grade (**Figure 3A**). CCT2 expression in BRCA and THYM was significantly different from tumor stage (**Figure 3B**). There was a significant difference in CCT2 expression between T1 and T 2 stages in BRAC and an increase in CCT2 expression with stage in PRAD (**Figure 3C**). No significant correlation between tumor stage classification and CCT2 expression was found in patients with other cancers.

### Relationship between CCT2 expression level and tumor immune cell infiltration level

**Figure 4:**
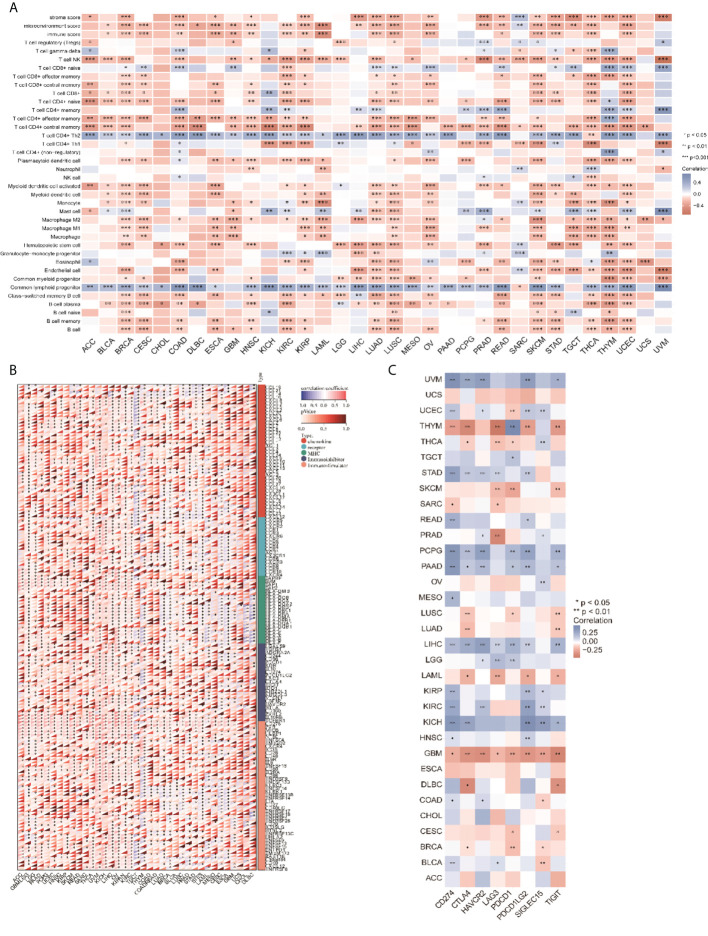
CCT2 expression and cancer immune correlation. **(A)** CCT2 expression levels in different tumors correlated with the infiltration levels of 26 immune-related cells. **(B)** CCT2 expression levels in different tumors correlated with immune genes. **(C)** CCT2 expression levels in different tumors correlated with immune checkpoints.

We analyzed the relationship between CCT2 expression levels and the infiltration levels of 26 immune-related cells. The results showed that the level of immune cell infiltration was significantly correlated with CCT2 expression in most cancer types, and CCT2 expression was negatively correlated with the level of most immune cell infiltration processes, but the CCT2 expression levels were negatively correlated with the level of T cell CD4+ Th2 and common lymphoid progenito cell infiltration (**Figure 4A**). In addition, we also analyzed the relationship between CCT2 expression and immune-related genes in 33 tumors. The results are shown in **Figure 4B**, where most of the immune genes were positively correlated with the CCT2 expression profile in 33 tumors. The immune checkpoint results showed that the CCT2 expression levels showed a positive correlation with the immune checkpoints in most tumors, except for THYM and GBM (**Figure 4C**, *p* < 0.05).

### Relationship between CCT2 gene expression and immune neoantigens, TMB, and microsatellite instability

**Figure 5:**
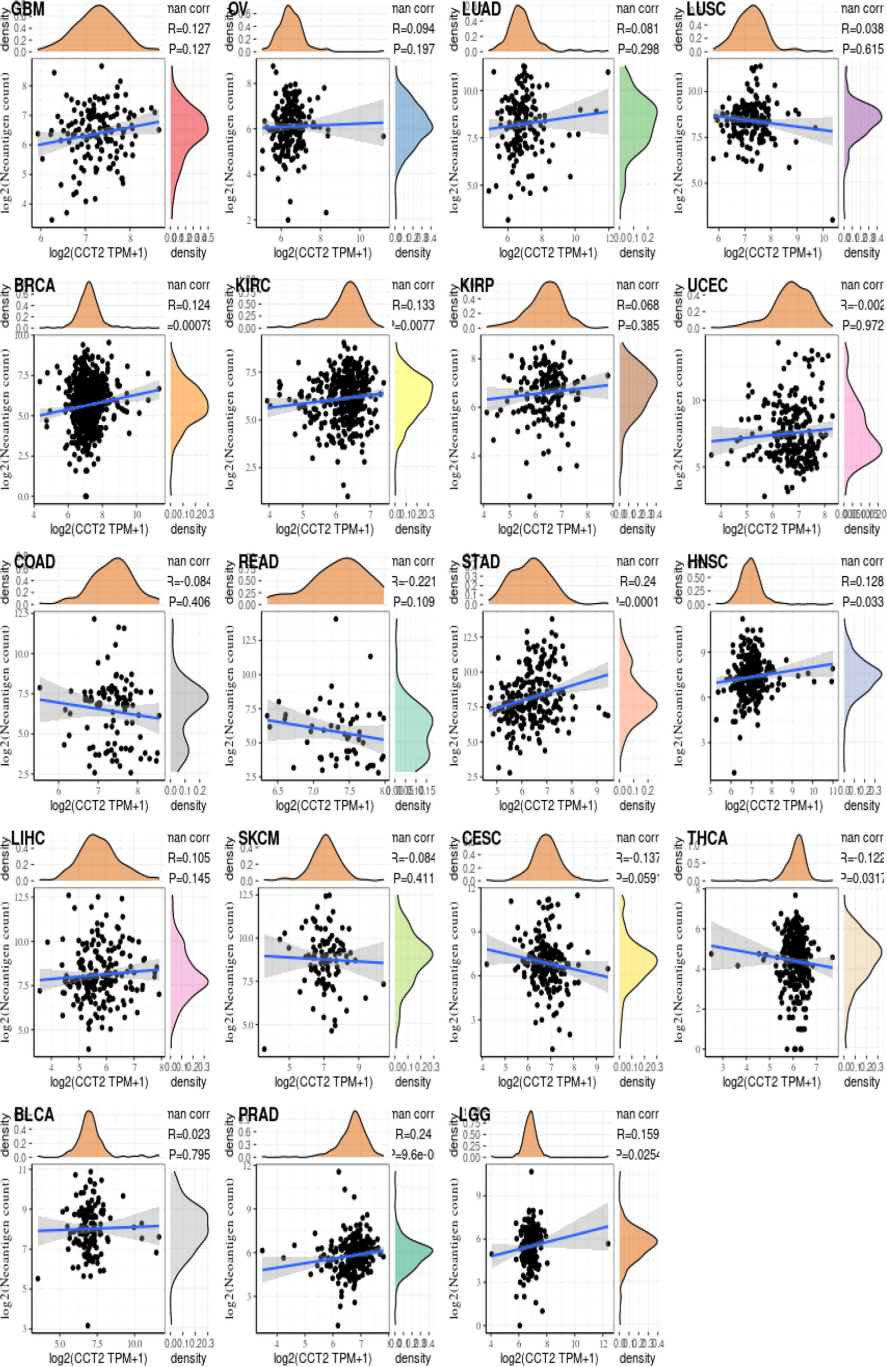
CCT2 expression correlates with immune neoantigens.

**Figure 6:**
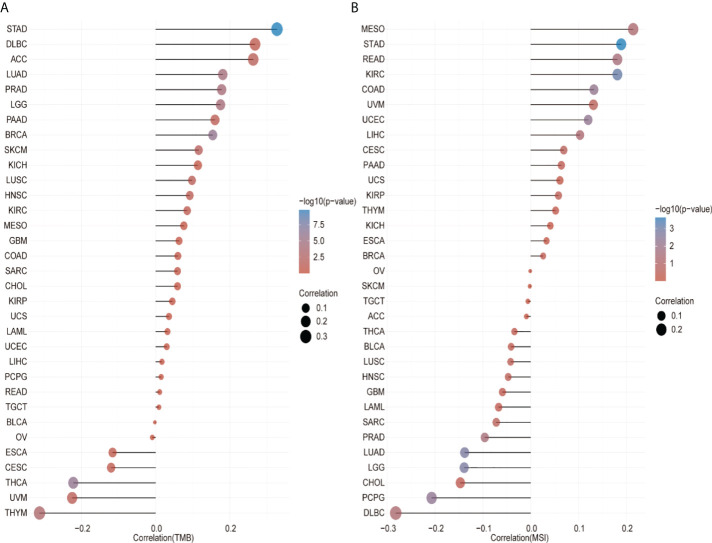
CCT2 expression correlates with tumor mutation burden (TMB) and microsatellite instability (MSI). **(A)** Lollipop plot of correlation between CCT2 expression and TMB level in pan-cancer analysis. **(B)** Lollipop plot of correlation between CCT2 expression and MSI level in pan-cancer analysis.

We analyzed the correlation between the CCT2 expression levels and immune neoantigens, TMB and MSI and found that all three were fundamentally associated with sensitivity to immune checkpoint inhibitors. **Figure 5** shows the correlation between CCT2 expression and tumor immune neoantigens. It was shown that immune neoantigens were significantly positively correlated with BRCA, KIRC, KIRP, STAD, HNSC, PRAD, and LGG and significantly negatively correlated with THCA. Considering that TMB and MSI play an important role in the process of tumor immunotherapy, we also examined the relationship of pan-cancer CCT2 expression with TMB and MSI, and the results are shown in **Figure 6**. CCT2 expression showed a positive correlation with TMB in most tumors and a negative correlation with BLCA, OC, ESCA, CESC, THCA, UVM, and THYM (**Figure 6A**). Similarly, CCT2 expression showed a positive correlation in MSI in most tumors and a negative correlation in BLCA, OC, ESCA, CESC, THCA, UVM, and THYM and in negative correlation in OV, SKCM, TGCT, ACC, THCA, BLCA, LUSC, HNSC, GBM, LAML, SARC, PRAD, LUAD, LGG, CHOL, PCPG, and DLBC (**Figure 6B**).

### GSEA analysis of the high and low expression of CCT2 gene in tumors

**Figure 7:**
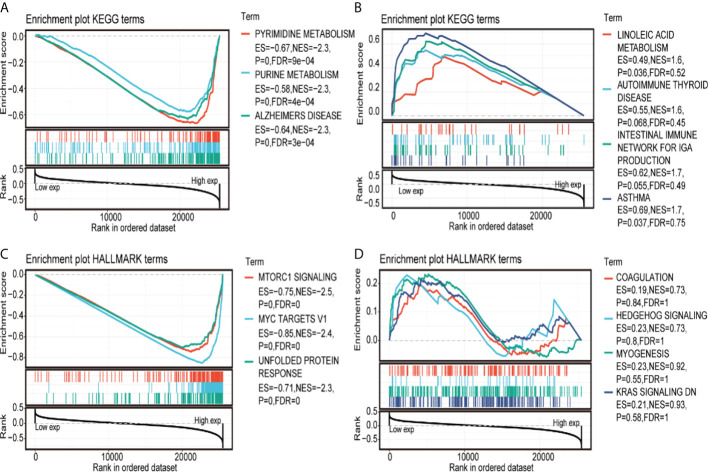
Gene set enrichment analysis (GSEA). **(A, B)** Kyoto Encyclopedia of Genes and Genomes annotation of CCT2 with GSEA. **(C, D)** HALLMARK annotation of CCT2 with GSEA.

To study the effect of gene expression levels on tumor, we classified the samples into high and low parts and analyzed the enrichment of the KEGG and HALLMARK pathways between the two groups of high- and low-expression part by GSEA; the results are shown in **Figure 7**. The most significant top three pathways are visualized as follows: CCT2 gene mainly loads PYRIMIDINE METABOLISM, PURINE METABOLISM, HEDGEHOG SIGNALING, and other pathways.

### External dataset validates the heterogeneous role of CCT2 in cancer

**Figure 8:**
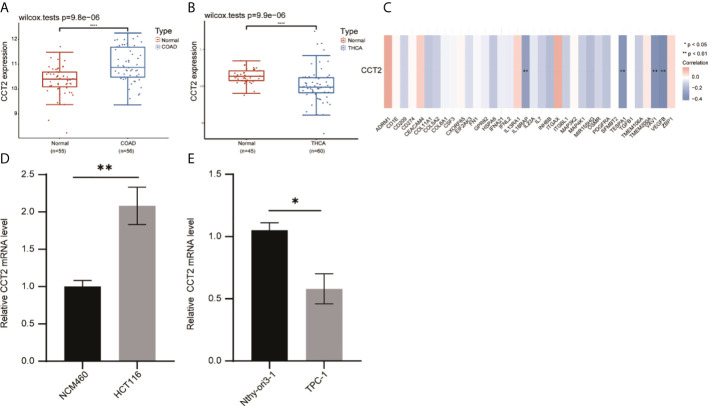
Validation of the correlation between CCT2 expression in tumors and immune genes. **(A)** Expression of CCT2 in colon cancer. **(B)** Expression of CCT2 in thyroid cancer. **(C)** Correlation between CCT2 expression and some immune genes in colon cancer. **(D, E)** RT-qPCR detection of CCT2 expression in colon cancer cells and thyroid cancer cells. *p < 0.05, **p < 0.01, ****p < 0.0001.

Meanwhile, in our published study, we found that Liu et al. (15) analyzed the expression and prognosis of CCT2 in various cancers and focused on the role played by CCT2 in breast cancer. In our study, we systematically analyzed the prognosis of CCT2 expression in various cancers and elaborated the correlation between CCT2 expression and tumor immunity. We also performed validation in the GEO dataset, which showed that CCT2 was highly expressed in colorectal cancer and less in thyroid cancer compared with normal tissue (**Figures 8A, B**). We also verified the correlation between CCT2 expression in colon cancer and immune genes (**Figure 8C**). Subsequently, we also examined the expression levels of CCT2 in colon cancer cells (HCT116), thyroid cancer cells (TPC-1), and normal cell lines (NCM460, Nthy–cri3-1). The results showed that CCT2 was highly expressed in colon cancer cells and less expressed in thyroid cancer cells (**Figures 8D, E**).

## Discussion

Human TCP1-containing chaperonin containing TCP1 subunit 2 (CCT2) is an isoform of heat shock protein 60 in eukaryotic cells that is involved in the metabolism of many cells and is highly expressed in many malignant tissues, where tumorigenesis, development, and prognosis are closely related (7, 12, 13). In our study, CCT2 was highly expressed in 27 tumors, and this was confirmed by IHC results. In other studies, the expression levels of TRiC subunits TCP1, CCT2, CCT 3, CCT 4, CCT 5, CCT 6A, CCT 7, and CCT 8 were significantly upregulated in HCC (14). PAAD, READ, STAD, and UCEC are significantly upregulated in CCT2 expression than in adjacent normal tissues (9, 15).

Our analysis of Kaplan–Meier survival of tumors by TCGA data displayed that a high CCT2 expression was related to poor prognosis in ACC, GBMLGG, LIHC, LUAD, MESO, SARC, and THYM. Similarly, one study reported that, in breast cancer, a high expression of CCT2 predicted its poor prognosis (15). In gallbladder cancer, it was shown by multifactorial analysis that a positive CCT2 expression was negatively correlated with low postoperative patient survival and positively correlated with high mortality (10). A study by Showalter et al. (16) overexpressed CCT2 by 1.3–1.8-fold in breast cancer cells by a lentiviral vector and found that cells overexpressing CCT2 were more aggressive and had a higher proliferation index, while CCT2 depletion in a homozygous mouse model of triple-negative breast cancer prevented tumor growth. Similarly, in a study by Guest et al. (17), they found that TCP1 and CCT2 are repeatedly altered in breast cancer and that TCP1 and CCT2 are required for the growth/survival of breast cancer cells *in vitro* and are determinants of overall survival in breast cancer patients. It has also been shown that TSPAN31 is highly expressed in gastric cancer (GC) tissues and that a high expression of TSPAN31 leads to a poor prognosis in GC patients. TSPAN31 regulates GC cell proliferation, migration, and apoptosis, and this regulatory mechanism is achieved by the co-expression of TSPAN31 (12). These data suggest that the CCT2 subunit is an important component of chaperone protein activity and is required for some tumorigenesis.

A growing body of proof support TMB as a possible biomarker of response to immune checkpoint inhibitors in most cancers (18, 19). These studies counsel that a better burden of nonsynonymous mutations in tumors promotes inflated neoantigen formation, creating tumors that are a lot immunogenic and therefore up to the clinical response to therapy (20, 21). Within the present study, we tend to evaluate the association between CCT2 and TMB and established that CCT2 was not related to most tumor TMBs, except STAD, SKCM, PRAD, LUAD, LGG, HNSC, COAD, CESC, BRCA, and THYM. Tumor immune infiltrating cells play a very important role within the immune regulation of tumor tissues (22, 23). An increasing range of studies has found that tumor immune infiltrating cells square measure closely related to immune checkpoint suppression and prognostic efficaciousness (24–27). To explore the connection between CCT2 expression and multiple infiltrating lymphocytes, we have a tendency to analyze the relative fraction of infiltrating immune cell sorts in 33 cancer sorts exploitation CIBERSORT. We found these associations again to be tumor type dependent. In addition, our enrichment analysis suggests that CCT2 can potentially influence cancer etiology or pathogenesis through HEDGEHOG SIGNALING. It has been shown that, during hypoxia in colorectal cancer, hypoxia activates the hedgehog pathway, CCT2 helps protein folding by binding to the oncogenic protein gli1, and a high expression of CCT2 and glii-1 enhances tumor invasion and migration *in vivo* and *in vitro* (28). The above-mentioned data suggest that CCT2 expression levels are closely associated with the immune infiltration of tumor cells and affect patient prognosis.

A fundamental challenge in the diagnosis and treatment of cancer is currently to detect changes in gene expression during tumorigenesis and progression and their relationship with prognosis. The heterogeneity of different molecular signaling pathways in tumor progression and postoperative recurrence in low- and high-grade tumors is a hallmark of bladder cancer, which is also useful in assessing tumor prognosis and patient survival outcomes. The comprehensive molecular characterization of multiple cancer types and corresponding patient clinical data collected through TCGA, the HAP database collecting protein expression levels in each tissue, and the XCELL database analyzes the correlation of gene expression with immune cell infiltration, thus providing the possibility of genomic studies of cancer.

In this study, we tend to analyze the expression of CCT2 in normal and neoplastic tissues. We also evaluated the prognostic worth of CCT2 in pan-cancer as supported by the TCGA dataset. Subsequently, we analyzed the relationship between CCT2 expression levels and immune cell infiltration among others. The results recommend that CCT2 can be considered an associate independent prognostic factor for multiple tumors, in which the CCT2 expression levels are completely different in several tumors and predict different prognostic outcomes; however, this requires investigating the particular role of CCT2 in every cancer in several tumors. CCT2 expression is associated with TMB, MSI, and immune cell infiltration. Its effect on tumor immunity also varies by tumor type. These findings may lead to the realization of a more precise and personalized immunotherapy in the future.

## Data availability statement

The original contributions presented in the study are included in the article/supplementary material. Further inquiries can be directed to the corresponding author.

## Author contributions

WL: conception and design,writing the article,data collection. LS: analysis and interpretation. JP: data collection, analysis and interpretation, writing the article. SW: writing the article, analysis and interpretation. All authors contributed to the article and approved the submitted version.

## Funding

This work was supported by the Natural Science Foundation of Gansu Province (20JR10RA724), the Second Hospital of Lanzhou University Cuiying Science and Technology Innovation Program Project (CY2020-MS06), and the Science and Technology Plan Project of Lanzhou City (2020-ZD-91).

## Conflict of interest

The authors declare that the research was conducted in the absence of any commercial or financial relationships that could be construed as a potential conflict of interest.

## Publisher’s note

All claims expressed in this article are solely those of the authors and do not necessarily represent those of their affiliated organizations, or those of the publisher, the editors and the reviewers. Any product that may be evaluated in this article, or claim that may be made by its manufacturer, is not guaranteed or endorsed by the publisher.
